# THE ROLE OF VIRTUAL PEER HEALTH SUPPORT IN ALTERNATIVE ILLNESS APPRAISALS AND PATIENT SELF-REAPPRAISAL AFTER CANCER

**DOI:** 10.1093/geroni/igac059.2241

**Published:** 2022-12-20

**Authors:** Gul Seckin

**Affiliations:** University of North Texas, Hickory Creek, Texas, United States

## Abstract

Technology is transforming how older adults find health-related support and get connected with others who cope with similar healthproblems. This study examined the relationship between virtual peer health support and alternative appraisals of illness experience in old age among people who use the Internet for cancer related support (N=157, Age Range: 50-79, Mean = 57). Regression models with interaction terms examined whether and to what extent virtual peer health support was associated with differential illness appraisal as well as and post-diagnosis self-reappraisal. Results demonstrated that a) perceived benefits of virtual peer health support is a significant predictor of self-reported self-reappraisal (β = .24, p < .01) b) appraisals of illness experience as a traumatic event and/or as a life challenge are both significantly associated with virtual peer health support (β = .18, p < .05 and β = .23, p < .01), and c) appraisal of illness experience as a personal growth growth opportunity is not significantly associated with virtual peer health support (β = .10, p < .23). These results suggest the statistical interactions of the virtual peer health support with illness appraisals are significant predictors of self-reappraisal post-diagnosis. Specifically, those who appraised cancer to have been a traumatic experience and/or life challenge for them perceived virtual peer support to be positively influential on their self-perception after cancer diagnosis, which is in contrast to those who perceived cancer experience as an opportunity for personal growth and thus possibly not deriving further significant additional benefits from virtual peer support.

